# Phenolic Compounds from Polygonum chinense Induce Growth Inhibition and Apoptosis of Cervical Cancer SiHa Cells

**DOI:** 10.1155/2020/8868508

**Published:** 2020-12-18

**Authors:** Wei Chen, XianMin Shen, Li Ma, Rong Chen, Qin Yuan, YunFeng Zheng, CunYu Li, GuoPing Peng

**Affiliations:** ^1^Suzhou TCM Hospital Affiliated to Nanjing University of Chinese Medicine, Suzhou 215109, China; ^2^School of Pharmacy, Nanjing University of Chinese Medicine, Nanjing 210023, China; ^3^Suzhou Institute for Food and Drug Control, Suzhou 215104, China

## Abstract

Cervical cancer is considered to be one of the most serious malignant tumors in women. Natural compounds have been considered as important sources in the search for new anticancer agents. Polygonum chinense (PC) has been used as herbal medicine and Chinese cool tea. By activity-guided of the extracts from PC, PC_water_ shows good growth inhibition on SiHa cell, then by chromatographic analysis (HPLC and HPLC-MS/MS), we found twelve components, seven were phenolic compounds (PHE), two PHE named ellagic acid and corilagin were found to show strong growth inhibition effects in SiHa cell dose-dependently, while the seven phenolic compounds showed low inhibition on the common human HcerEpic cell. Further research found ellagic acid and corilagin induced G2 phase cell cycle arrest by upregulating levels of P53, Bcl-2, caspase 3, and caspase 9, while the Bax was reduced. These results suggested that PHE from PC might have potential anticancer effects against SiHa cells by acting through the apoptosis pathway, PHE from PC might have the potential to be used as a nutraceutical for the prevention and treatment of ovarian cancer.

## 1. Introduction

Cervical cancer is the second most common female cancer worldwide [1]. There are more than half a million new cases and more than 200,000 deaths each year [2, 3]; in recent years, the incidence of cervical cancer in China is on the rise, and the incidence of cervical cancer tends to be younger [4, 5]. It is generally accepted that radical surgery or radiotherapy can be curative for the majority of patients with early-stage cervical cancer, while for patients with advanced cervical cancer whose prognosis is still poor, chemotherapy or neoadjuvant chemotherapy is always the first choice [6]. Therefore, many researchers have been trying to find more effective chemotherapy drugs to treat cervical cancer cells. Apoptosis is an active form of cell suicide controlled by a network of genes and it is an essential process, as well as a key role, in the pathogenesis of diseases including cancer [7]. Agents that suppress the proliferation of malignant cells by enhancing apoptosis may represent a useful mechanistic approach to both cancer chemoprevention and chemotherapy [8, 9]. However, unfavorable side effects and resistance to many currently used anticancer agents are serious problems. So we should develop more safe and effective therapeutic agents for cancer treatment [10].

Polygonum chinense (PC), the overground part of Polygonum Chinese Linn., is mainly distributed in Guangdong, Guangxi, Fujian, Hainan, and other places. Despite this, it was usually used for Chinese cool tea, and it is also a traditional Chinese medicine [11]. PC contains phenolic compounds (PHE), flavonoids, and other chemical components. It has a variety of biological activities, like antitumor and antioxidant [12]. Existing studies have shown that phenolic acid has an antitumor effect [13–15], for example, PHE of tetrastigma antihuman hepatoma [16], PHE from Rubus fairholmianus antihuman breast tumor [17], and Betulinic acid in the bark of birch trees anticervical epithelial tumor [18]. Studies have found that PC has antitumor activity in liver cancer and colorectal cancer [19]. However, the anticervical tumor effect of PC has not been reported. Cervical epithelial tumor contains a variety of bacteria and viruses-associated factors, among which including the HPV virus is one of the main pathogenic factors. Cervical carcinoma in situ is induced by HPV through the degradation of oncogene E6E7 and P53 protein in cervical epithelial cells and blocking the normal suppressor P53 pathway [20, 21].

In this study, cervical squamous epithelial cell SiHa was used as the research object, to examine the inhibitory of PC_water_ extract and the phenolic acids on SiHa cell proliferation, in order to find the active PHE with antitumor.

## 2. Materials and Methods

### 2.1. Plant Material and Seven Phenolic Compounds

Polygonum chinense was collected from Pingyang county, Guangxi province, China. The samples were identified by Professor Xunhong Liu to be the dried whole grass of Polygonum Chinese Linn. The samples were stored in a dry, dark room at the School of Pharmacy, Nanjing University of Chinese Medicine.

Chebulagic acid, citrate acid, gallic acid, chlorogenic acid, brevifolin carboxylic acid, corilagin, and ellagic acid (Shanghai PureOne Bio Tech Co., Ltd.) are stored in dry, dark, 2-8°C.

### 2.2. Extractions of PC

The water extract of PC (PC_water_) was prepared by boiling PC crude materials (50 g of fine powder) with water (500 mL, 1 h) at 100°C, extracted twice. Likewise, 50 g PC powder was extracted with ethanol (500 mL, 1 h) at 80°C, extracted twice to obtain the ethanol extract (PC_EtOH_). To prepare PC dichloromethane extract (PC_CH2Cl2_), 50 g of PC powder was boiled at 50°C (500 mL, 1 h) with CH_2_Cl_2_, extracted twice [22]. The fractions were concentrated in a vacuum and then freeze-dried to obtain loose fine powder. HPLC chromatograms of different PC extracts (PC_water_, PC_EtOH_, and PC_CH2Cl2_) were carried out using a Waters Series 2695 liquid chromatography (Waters Technologies, Milford, MA, USA). A Boston RP C18 column (250∗4.6 mm, 5 *μ*m) was used. Samples were separated using a gradient mobile phase consisting of 0.2% (*v*/*v*) formic acid water (A) and acetonitrile (B). The gradient conditions were 5–65% B at 0–45 min. The flow rate was set at 1.0 mL/min. The detection wavelength was 365 nm. The sample concentration was 1 mg/mL, and the injection volume was 10 *μ*L.

### 2.3. Cell Culture

The SiHa cell line was obtained from American Type Culture Collection (ATCC, Manassas, Virginia, VA, USA); HcerEpic cell was obtained from ScienCell (San Diego, Los Angeles, LA, USA). The cells were maintained in medium RP1640 containing 10% FBS, high glucose DMEM cultured with 100 U/ml of penicillin, and 100 U/ml of streptomycin, in a humidified CO_2_ (5%) incubator at 37°C. All reagents for cell cultures were purchased from Invitrogen (Carlsbad, California, CA, USA).

### 2.4. Antiproliferative Activity Assay

SiHa and HcerEpic cells were cultured in 96-well plates at approximately 7.5 × 103 cells per well and incubated for 12 h. Then, cells were treated with different concentrations of PC extracts (5, 10, 25, 50, and 100 *μ*g/mL) or seven PC PHE (10, 20, 40, 60, and 80 *μ*M). After incubation of 48 h, then 10 *μ*L CCK8 reagent added per hole, placed in the incubator 2 h, incubation, enzyme standard instrument determination at 450 nm absorbance (OD value).

#### 2.4.1. Cell Vitality

Cell vitality∗(%) = [A (dosing) − A (blank)]/[A dosing (O) − A (blank)] × 100%A (dosing): OD values of Wells with cell, CCK8 solution, and drug solution.

A (blank): OD values of Wells with medium, CCK8 solution, and no cells.

A (0 dosing): OD values of Wells with cell and CCK8 solution but no drug solution.

### 2.5. Morphological and Differentiation Analysis

According to the cell inhibition rate spread 96 orifices and dosing method, after dosing in 37°C, 5% CO_2_ constant temperature incubator culture supernatant after 48 h to refuse to cells, PBS cleaning after 1 time and 4% paraformaldehyde-fixed at room temperature for 15 min, abandon paraformaldehyde PBS cleaning after 2 times, add 0.5 ml Hoechst 33258 dyeing liquid, avoid light 15 min after incubation, and abandon the dye absorption and PBS were observed under inverted microscope after cleaning.

### 2.6. Cell Cycle Analysis

The DNA content and cell cycle distribution of SiHa cells were determined by flow cytometry. Cell plated at a density of 5 × 10^5^ per well in 6 well plates was treated with ellagic acid and Corilagin and harvested at 48 h. The cells were washed once in PBS. They were then fixed in cold 70% ethanol and stored at 4 uC for 30 min [23]. Then, ethanol was removed and the cells were resuspended in PBS. The fixed cells were then washed with PBS, treated with RNase (100 mg/ml), and stained with Propidium Iodide (PI, 20 mg/ml) in the dark for 30 min at 37 uC. The cell cycle was analyzed by flow cytometry (BD Biosciences, Franklin Lakes, NJ) and analyzed by Flowjo software.

### 2.7. Western Blot Analysis

We followed the Western blot methods of Tu et al. 2020 [24]. SiHa cells cultured in 100 mm dishes were treated with the required ellagic acid and corilagin concentration of 25, 50, and 75 *μ*M for 48 h. The culture was terminated after 48 h. Then, cells were collected and proteins were extracted with RIPA lysis buffer containing a protease inhibitor cocktail. The total protein concentrations were measured using the Bradford method, and the normalized protein samples were added to 4 sample buffer, then boiled and denatured. Equal amounts of proteins were separated by SDS-PAGE and then transferred to nitrocellulose membranes. The membranes were blocked and then probed with indicated primary antibodies, respectively, with anti-P53 (1 : 1000), anti-Bax (1 : 1000), anti-Bcl-2 (1 : 1000), anti-caspase 3 (1 : 2500), anti-caspase 9 (1 : 2500), anti-cleaved-caspase 3 (1 : 1000), and anti-cleaved-caspase 9 (1 : 1000), at 4°C overnight. Antibodies were purchased from Abcam (Santa Cruz, California, CA, USA); others were from Cell Signaling Technology (Cell Signaling, Danvers, Massachusetts, MA, USA). All antibodies were diluted with 5% BSA in TBST buffer. The blots were rinsed and then incubated with secondary antibodies (anti-mouse antibody or anti-rabbit antibody, 1 : 5000, Cell Signaling Technology). Reactive bands were visualized using ECL (Thermo Fisher Scientific; Waltham, MA, USA) and then calibrated by ChemiDoc Imaging System (Bio-Rad; Hercules, California, CA, USA).

### 2.8. Statistical Analysis

The significant difference among groups was statistically performed by one-way analysis of variance (ANOVA) combined with Tukey's test by SPSS v. 22.0 program (IBM Corp., Armonk, New York, NY, USA). Probability value less than 0.05 (*p* < 0.05) was considered to be statistically significant. Data are expressed as the means_standard error of mean (SEM) of at least three independent experiments.

## 3. Results

### 3.1. PC Extracts Inhibited SiHa Cells

**Figure 1:**
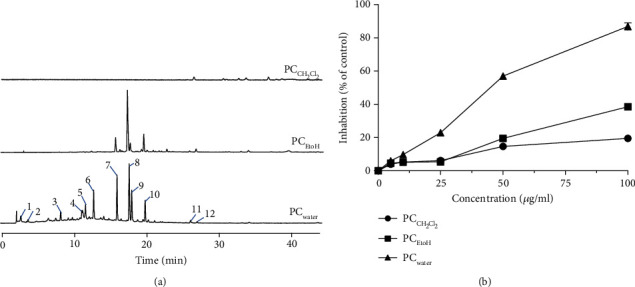
Effects of different extracts of PC in antiproliferation activities in SiHa cells. (a) HPLC chromatograms of different extracts, all at 1 mg/mL, from PC, PCwater (water extract of PC), PCEtOH (EtOH extract of PC), and PCCH2Cl2 (CH2Cl2 extract of PC). (b) SiHa cells were treated with different extracts of PC or with a medium of 0.1% DMSO (dimethylsulphoxide) for 48 h, after which cells were counted under CCK8 assay.

Water, 95% alcohol extraction solution, and dichloromethane extraction solution were analyzed by High-performance liquid chromatography (HPLC) of extraction solution and showed the difference in composition (Figure 1(a)). The HPLC results showed that the water extraction of PC contained the most components, the ethanol sample was similar to the water one, and the dichloromethane contained the least components. The content of components in the water extract was higher than that of ethanol. To a certain extent, differences in chemicals might cause possible differences in their biological capacities.

To investigate the effect of PC extracts, cultured SiHa cells were treated with different extracts at indicated concentrations for 48 h. As shown in (Figure 1(b)), PC_Water_ inhibited the proliferation of SiHa cells with an IC50 value of 48.7 ± 2.5 *μ*g/mL, whereas PC_EtOH_ and PC_CH2Cl2_ exhibited little effects (IC50 > 100 *μ*g/mL). Similar to antiproliferative activity, PC_water_ showed little morphological changes towards cultured SiHa cells. These results suggested that PC_water_ extract could be responsible for the antiproliferation of SiHa cells.

The water extract was elucidated on the basic of ESI-Q-TOF/MS (Table 1). Combine the components and HPLC chromatogram, it can be concluded that the water extract of carbonaceous charcoal is basically phenolic substances, and that the proportion of phenolic substances is more than 70%.

### 3.2. Validation of Cell Viability of Several Acidic Components of PC

**Figure 2:**
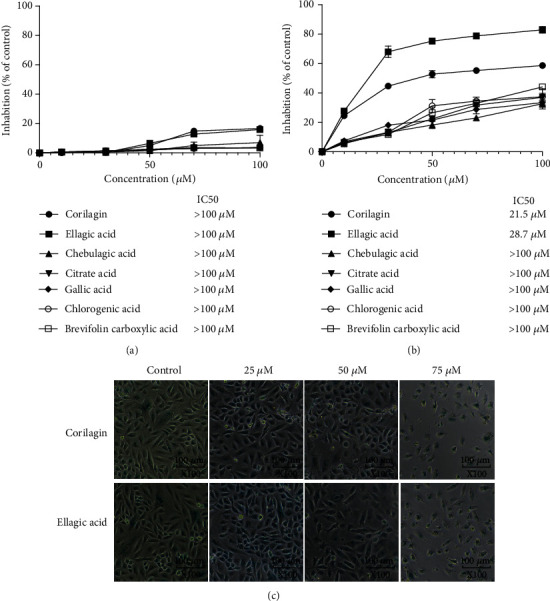
(a) Antiproliferation activity of PC phenolic on HcerEpic cells. HcerEpic cell was treated with 7 PHE at different concentrations or with medium (0.1% DMSO) for 48 h, after which the cells were assayed under CCK8 assay. IC50 value was calculated using SPSS statistics software. (b) Antiproliferation activity of PC phenolic on SiHa cells. SiHa cell was treated with 7 PHE at different concentrations or with medium (0.1% DMSO) for 48 h, after which the cells were assayed under CCK8 assay. IC50 value was calculated using SPSS statistics software. (c) Morphological of SiHa cell treated with corilagin and ellagic acid (25 *μ*M, 50 *μ*M, and 75 *μ*M).

CCK8 cell viability test of 7 acidic substances showed that all acids had low inhibitory effect on HcerEpic cells, and IC50 was all above 100 *μ*M (Figure 2(a)), while ellagic acid and corilagin had a good inhibitory effect on SiHa cell proliferation (Figure 2(b)). After the treatment of SiHa cells with different concentrations of ellagic acid and corilagin for 48 h, the cells in the control group grew adherent to the wall with a large number, regular cell morphology, clear cell membrane, and uniform refraction, as shown in Figure 2(c). The most active component was ellagic acid with an IC50 was 21.5 *μ*M. While compound corilagin IC50 was 28.7 *μ*M, the other PHE possessed low activities of cell inhibition. Experimental cell number decreases with the increase of drug concentration, the shape is irregular, refraction sex is reduced, and cell shrinkage, collapse, and debris, visible when the concentration of ellagic acid and corilagin is 75 *μ*m/L, the cells lose their original form and most of the disintegration of cellular debris, floating in the culture bottle, according to the results of ellagic acid can inhibit the growth of SiHa cells, corilagin also agree with CCK8 results, and the inhibitory effect is more obvious with the increase of drug concentration.

### 3.3. Experimental of SiHa Cell Apoptosis Induced by Ellagic Acid and Corilagin

**Figure 3:**
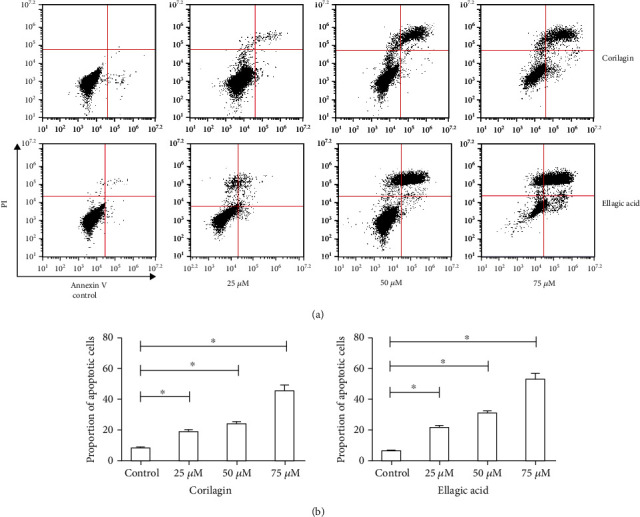
Effects of ellagic acid and corilagin on apoptosis of SiHa cell. (a) Induction of apoptosis of SiHa cell after ellagic acid and corilagin treatment. SiHa cell was treated with ellagic acid and corilagin at doses of 0, 25, 50, and 75 *μ*M for 48 h. Apoptosis was measured by flow cytometry. (b) Statistical analysis of the percentages of the apoptotic cells. Data shown were representatives of three experiments.

The effects of ellagic acid and corilagin on cell cycle distribution were evaluated by flow cytometry. When ellagic acid and corilagin were administered at the dose of 50 *μ*M, SiHa cells exhibited increased cell percentages in G2 phase with an increase of SiHa cells from 3.50% to 12.18%. To further investigate whether ellagic acid and corilagin could induce apoptosis of the cell, the apoptotic cell percentages were analyzed by flow cytometry. SiHa cell was treated with different concentrations of ellagic acid and corilagin (0, 25, 50, and 75 *μ*M) for 48 h. The percentages of apoptotic cells were significantly increased in the treated group compared to the control group (*p* < 0.05) (Figure 3) for both cell lines in a dose-dependent manner. The apoptotic cells increased from a total of about 10% to 70% for SiHa cells. Taken together, ellagic acid and corilagin treatment could induce SiHa cell apoptosis and G2 phase arrest.

### 3.4. Western Blot Detected the Expression of Proteins Related to SiHa Cell Apoptosis

**Figure 4:**
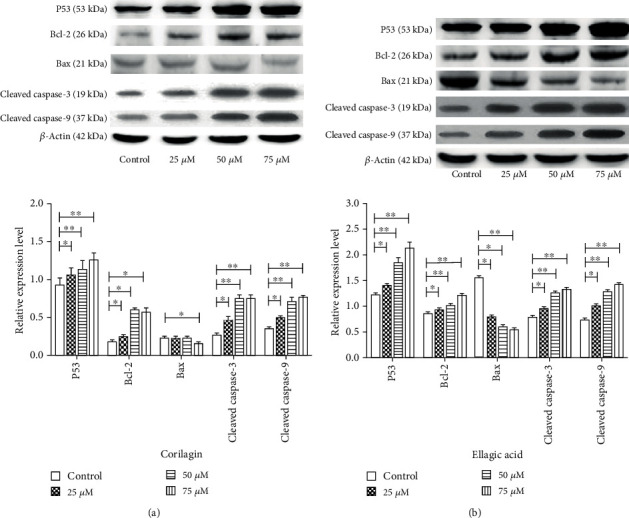
Ellagic acid and corilagin induce the P53 signaling pathway in SiHa cells. Cells were treated with various concentrations of selected ellagic acid and corilagin of 25, 50, and 75 *μ*M for 48 h, after which protein expression in total cells was assayed by Western blot analysis. (a) Ellagic acid-induced protein expression, (b) corilagin induced protein expression. The relative abundance of each band was quantified, and the control levels were set at 1.0 (lower panel). Data are expressed as percentage of control, in means ± SEM (*n* = 3).^∗^*p* < 0.05, ^∗∗^p < 0.01, compared with control.

After SiHa cells were treated with different concentrations of ellagic and corilagin for 48 h, the expression levels of P53, Bax, Caspase3, and Csapase9 proteins were significantly upregulated, and the expression levels of Bcl-2 were downregulated compared with the control group (Figure 4), and the differences were statistically significant.

## 4. Discussion

In this study, we found for the first time that water extraction of PC has a significant inhibitory effect on the SiHa cells. After the analysis of the water extraction, we further found water extraction of PC was mainly composed of phenolic acids, in which 12 compounds (including seven phenolic acids) were identified. The inhibitory rate of the identified phenolic acids on HPRPEC cells was low, while ellagic acid and corilagin had a significant inhibitory effect on SiHa cells. Furthermore, ellagic acid and corilagin induced cell cycle arrest at the G2 phase. Finally, we found ellagic acid and corilagin can upregulate the protein expression levels of P53, Bcl-2, and caspase3/9 and downregulate the protein expression level of Bax in SiHa cells, thus, inducing cell apoptosis.

It has been estimated that 30%-40% of cancers can be prevented by dietary and lifestyle conditions [33]. Some anticancer drugs currently used in clinics, such as paclitaxel and camptothecin, are derived from natural products. Thus, the search for natural products with anticancer activity represents an interesting area, probably due to its diversity and unique mechanism of action [34]. PHE, widely found in herbal medicines, is a kind of compound with potential antitumor activity. For example, polyphenols have been shown to be cytotoxic effects on tumor cells [35].

There are a lot of phenolic acids and flavones in PC, flavonoids ingredients are recognized the antitumor active material, and this research discovered for the first time Chinese knotweed water extraction liquid of cervical cancer has good pharmacological activities, found main ingredients for its further research for phenolic acids 12 compounds, including ellagic acid and ko lira Beijing for phenolic hydroxyl tannins, structure similar to that of the two phenolic acids lower toxicity to normal cervical epithelial cells and the activity of SiHa cells. Ellagic acid has previously been shown to be active in cervical cancer [36, 37], and Corrilla has also been found to be active in some tumors [38]. Bcl-2 and Bax are two key proteins in the apoptotic pathway, and downregulation of Bax by upregulating Bcl-2, p53, and Caspase-3/9 can activate the apoptotic pathway. This study found that the apoptosis of SiHa in cervical epithelial tumor cells induced by two polyhydroxyphenolic acids in the mother carbon was realized through this pathway.

In conclusion, PC water, ellagic acid, and corilagin have good inhibitory effects on SiHa in vitro, and it was first found that PC_water_ and its PHE corilagin have good cytotoxic effects on SiHa. However, whether ellagic acid and corilagin can be used in combination with surgery, chemoradiotherapy is for clinical auxiliary. Further in vitro and clinical trials are needed to assist the treatment of cervical cancer. In addition, the composition analysis of PC is not complete at present, and other phenolic acids with similar structure should be further analyzed. The anticervical cancer effect of PC was further studied in vivo.

## 5. Conclusions

In summary, this study first presents the evidence that the water extract of PC has potential antiproliferation activity on SiHa cells. PHE were demonstrated to be the active constituents of PC extract. These PHE have different backbone structures, and thus each has distinct activity to inhibit the growth of cancer cells. The results of this study provided a molecular basis for the utilization of polar extract of PC and lay a foundation for further development of the identified bioactive PHE for the prevention and/or treatment of cervical cancer.

## Figures and Tables

**Table 1:** UHPLC-QTOF-MSMS identified in the typical base peak chromatogram (BPC) of PC_Water_ samples.

Peak no.	Retention (min)	Molecular formula	Measured mass m/z (error, ppm)	MS/MS fragments ions	Identification
1	2.823	C14H12O11	355.0311 (-0.61)	193.0139, 163.0401, 179.0721	Chebulagic acid [25]
2	3.841	C_7_H_12_O_6_	191.0561 (-1.51)	123.1132, 138.2661, 162.1097	Citrate acid [26]
3	8.461	C7H6O5	169.0144 (-0.91)	125.0252, 117.0357, 103.0188	Gallic acid [25]
4	11.499	C16H18O9	353.0875 (0.87)	191.0572, 135.0458, 155.0352	Chlorogenic acid [27]
5	11.896	C13H8O8	291.0150 (-1.23)	247.0265, 191.0361, 219.0310	Brevifolin carboxylic acid [28]
6	13.045	C_27_H_22_O_18_	633.0742 (-1.42)	481.0373, 301.9995, 169.0251	Corilagin [29]
7	16.314	C21H20O12	463.0888 (1.20)	301.0533, 179.0153, 125.0256	Quercetin-3-O-galactoside [25]
8	18.014	C14H6O8	301.0002 (-4.02)	145.0308, 117.0357, 173.0249	Ellagic acid [25]
9	18.375	C_15_H_12_O_8_	319.0475 (-1.9)	217.1129, 245.0381, 213.0478	Dihydromyricelin [30]
10	20.255	C21H20O11	447.0931 (0.41)	301.0060, 179.0000, 121.0306	Quercitrin [31]
11	26.613	C_22_H_18_O_13_	489.0674 (0.1)	299.3215, 271.0915, 125.0032	Enyssoside [32]
12	27.540	C_16_H_10_O_8_	329.0306 (-0.9)	298.1564, 270.1088, 314.0599	3,3′-Dimethyl ellagic acid [25]

Error (ppm): the difference between experimental mass and theoretical mass of the compound; indicated compound 8 undetected in PC sample; the compounds 1, 2, 3, 4, 5, 6, 8, and 10 were confirmed by chemical reference standards.

## Data Availability

The data used to support the findings of this study are included within the article.
